# Higher plasma IL-6 and PTX3 are associated with worse survival in left heart failure with pulmonary hypertension

**DOI:** 10.1016/j.ahjo.2022.100190

**Published:** 2022-08-10

**Authors:** Sara Helleberg, Adam Engel, Salaheldin Ahmed, Abdulla Ahmed, Göran Rådegran

**Affiliations:** a Department of Clinical Sciences Lund, Cardiology, Lund University, Sweden; b The Haemodynamic Lab, Section for Heart Failure and Valvular Disease, VO. Heart and Lung Medicine, Skåne University Hospital, Lund, Sweden

**Keywords:** Inflammation, Heart transplantation, Biomarkers, Left heart disease, Haemodynamics, Interleukin 6, Pentraxin related protein 3

## Abstract

**Introduction:**

Left heart failure (LHF) is commonly complicated by pulmonary hypertension (PH), increasing morbidity and mortality. The present study aimed to evaluate the prognostic value of inflammatory proteins in LHF with PH (LHF-PH).

**Materials and methods:**

The levels of 65 plasma proteins, analysed with proximity extension assay, were compared between healthy controls (*n* = 20), patients with LHF-PH (*n* = 67) comprising both HFpEF-PH (*n* = 31) and HFrEF-PH (*n* = 36), and in a LHF subpopulation before and after heart transplantation (HT, *n* = 19). Haemodynamic parameters were measured using right heart catheterization.

**Results:**

Plasma levels of Interleukin 6 (IL-6) and Pentraxin related protein PTX3 (PTX3) were elevated in LHF-PH vs. controls (*p* < 0.001), and these decreased after HT compared to before HT (p < 0.001). Plasma IL-6 and PTX3 correlated to elevated NT-proBNP (*r* = 0.44, *p* = 0.0002 and r = 0.4, *p* = 0.0009, respectively). Additionally, IL-6 correlated with mean pulmonary arterial pressure (*r* = 0.4, *p* = 0.0009) and mean right atrial pressure (*r* = 0.51, *p* < 0.0001). Higher levels of IL-6 and PTX3 were associated with worse survival rates in patients with LHF-PH (Log rank *p* < 0.01).

**Discussion:**

In patients with LHF-PH, higher plasma levels of IL-6 and PTX3 were associated with worse survival rates. Future larger studies to validate and investigate the direct clinical applicability of IL-6 and PTX3 as potential prognostic biomarkers are encouraged.

## Highlights

• Inflammation is related to heart failure and pulmonary hypertension progression.
• Plasma IL-6 & PTX3 were elevated in left heart failure and pulmonary hypertension.
• Elevated IL-6 & PTX3 decreased post heart transplantation towards controls' levels.
• Plasma IL-6 & PTX3 correlated to haemodynamics in patients with heart failure.
• Higher plasma IL-6 & PTX3 are related to worse transplantation free survival.

## 1 Introduction

Worldwide, 64.3 million people are estimated to live with heart failure (HF), a condition with a rising prevalence due to increased ageing and survival of cardiovascular diseases [1]. Complications of HF are also increasing, such as pulmonary hypertension (PH) which impacts negatively on morbidity and mortality. PH is divided into five subgroups, where group 2 PH is the most common, encompassing approximately 65–80 % of all cases of PH [2]. PH due to left heart disease (PH-LHD) can be caused by HF with preserved ejection fraction (HFpEF), HF with reduced ejection fraction (HFrEF), valvular heart disease and/or congenital heart disease [3]. Patients with PH-LHD have more severe symptoms and worse prognosis compared to those with LHD without PH [4].

PH is caused by passive backward transmission of increased end diastolic pressure in the left ventricle caused by systolic or diastolic dysfunction. This leads to interstitial fibrosis, reduced compliance and impaired contractility of the left ventricle [3,5]. Persistent pressure elevation in the pulmonary circulation causes hypoxia, pulmonary vasoconstriction as well as leakage from blood vessels which impairs gas exchange [3] and attracts inflammatory cells [5]. This leads to medial hypertrophy, intimal fibrosis [5] and collagen depositions in the pulmonary vessels [3]. These pressures further increase with exercise which worsen the symptoms [5].

Inflammation is activated as a protective response to increased stress in acute heart injury. However, it can evolve into chronic inflammation which activates basal remodeling and fibrosis driving HF progression. Previous studies have demonstrated that levels of inflammatory markers correlate with the severity and prognosis in HF [6,7]. Inflammation in HF is also thought to involve many different pathways and may partly explain why therapies targeting inflammation have not yet proven beneficial [6].

Inflammation is one of the major pathological pathways in HF and may thus be a useful target for the development of new diagnostic tools [8]. This was urged for in the task force of the sixth World Symposia on PH [9] and can be achieved by using biomarkers. Biomarkers offer a non-invasive means in aiding clinical decisions and may play a role in determining disease stage and treatment response. Furthermore, biomarkers can be used in treatment follow-up as well as to identify patients at high risk of morbidity and mortality [8,10]. The present study therefore aimed to investigate the role of inflammatory plasma proteins as prognostic biomarkers in patients with LHF-PH.

## 2 Materials and methods

### 2.1 Study population

The present study included 20 controls as well as 67 patients with pulmonary hypertension due to left sided heart failure (LHF-PH) of whom 19 were heart transplanted (HT). Patients with missing haemodynamics (*n* = 3) and unconfirmed PH diagnosis (*n* = 2) were excluded before analysis. Patients with PH after HT (*n* = 1) were excluded from pre- and postHT groups to only be included in LHF-PH. All participants were ≥ 18 years of age and provided written informed consent before enrolment. The study was performed in accordance with declarations of Helsinki and Istanbul and approved by the regional ethical board in Lund, Sweden (Dnr: 2010/114, 2010/442, 2011/368, 2011/777, 2014/92, and 2015/270).

### 2.2 Proteomic analysis

Venous plasma samples were collected from all participants between October 2011 and February 2017 and stored at −80 degrees Celsius in Lund Cardiopulmonary Registry (LCPR), a cohort of Region Skåne's biobank, established by Göran Rådegran September 2011. All plasma samples were collected from non-fasting participants. Sixty-five inflammatory proteins as well as NT-proBNP, were chosen from Proseek Multiplex immunoassay reagent kits (Cardiovascular II, III and Oncology II panel, Olink Proteomics, Uppsala, Sweden) and analysed with proximity extension assay (PEA) when blood samples were retrieved in March 2017.

PEA uses coupled antibodies that bind to their protein target, crosslinking with other similar antibodies and builds individual DNA-sequences for their target protein. Next, qPCR is used to amplify the sequences corresponding to each protein, to quantify the proteins´ levels [11]. The proteins are expressed in arbitrary units (AU) on a linear normalized protein expression scale, which corresponds to the inverted Ct-value of the qPCR.

PH in patients with LHF was defined as resting mean pulmonary arterial pressure (mPAP) ≥25 mmHg and was diagnosed by experienced cardiologists by right heart catheterization (RHC). A post-capillary PH was defined by a pulmonary arterial wedge pressure (PAWP) >15 mmHg, which was further classified into i.) isolated post-capillary PH, indicated by a pulmonary vascular resistance (PVR) ≤3 wood units (WU) and/or diastolic pulmonary pressure gradient (DPG) <7 mmHg or ii.) combined post- and pre-capillary PH, indicated by a PVR >3 WU and/or DPG ≥7 mmHg.

### 2.3 Right heart catheterization

During RHC, patients were placed in supine position. A Swan-Ganz catheter was inserted into the right internal jugular vein and further into the right cardiac atrium, ventricle and pulmonary artery. Cardiac output (CO) was measured using thermodilution. Mean right atrial pressure (MRAP), PAWP, systolic pulmonary arterial pressure (sPAP), diastolic pulmonary arterial pressure (dPAP), mPAP, arterial oxygen saturation (SaO_2_), mixed venous oxygen blood saturation (SvO_2_) and mean arterial pressure (MAP) were measured. From these parameters, the following formulas were used to calculate more parameters: cardiac index (CI) = CO/body surface area, stroke volume (SV) = CO/heart rate, stroke volume index (SVI) = CI/heart rate, DPG = dPAP-PAWP, transpulmonary pressure gradient (TPG) = mPAP-PAWP, pulmonary arterial compliance (PAC) = SV/(sPAP-dPAP), PVR = TPG/CO, left ventricular stroke work index (LVSWI) = (MAP-PAWP)*SVI, right ventricular stroke work index (RVSWI) = (mPAP-MRAP)*SVI. Measured haemodynamic parameters are presented in supplementary Table 2. Creatinine based estimated glomerular filtration rate was calculated using the revised Lund-Malmö formula [12].

### 2.4 Statistical analysis and study setup

The study setup is visualized in Fig. 3. Statistical analysis was performed using Graph pad prism version 9.1.2. Distribution of continuous data was analysed using Histograms. Since no normal distribution was found for any protein, continuous data were presented as median (interquartile range) and further analyses were performed using non-parametric tests. To visualize protein levels between groups, controls, LHF-PH, Pre-HT and Post-HT, boxplots were used. Mann-Whitney and Wilcoxon singed-rank tests were performed and a following two-stage step-up method of Benjamini, Krieger and Yekutieli of false discovery rate (Q = 0.01) was used to correct for mass-significance. Proteins were excluded if they did not meet three inclusion criteria after FDR, (I) significant change between controls and LHF-PH, (II) significant change between pre- and post-HT and (III) Post-HT-levels developing towards the protein levels of controls. Included proteins were correlated to N-terminal-pro-brain natriuretic peptide and eight haemodynamic parameters i.e. mPAP, PAWP, MRAP, SVI, CI, PVR, LVSWI and PAC, followed by FDR (Q = 0.01). To visualize the prognostic value for each potential biomarker, proteins with significant correlations were analysed using ROC-curves followed by survival analysis with Kaplan Meier based on patient data before August 2020. Due to the low number of events, multivariate analysis was not performed. Mann Whitney singed rank tests were used to analyse and examine impact of angiotensin converting enzyme inhibitors (ACEi), diabetes mellitus (DM) and hypertension (HTN) on the protein levels.

### 2.5 Proteins

The 67 inflammatory proteins analysed comprised Annexin A1, Azurocidin, C—C motif chemokine 2 (CCL2), C—C motif chemokine 3 (CCL3), C—C motif chemokine 15 (CCL15), C—C motif chemokine 16 (CCL16), C—C motif chemokine 17 (CCL17), C—C motif chemokine 22 (CCL22), C—C motif chemokine 24 (CCL24), CD27 antigen (CD27L), CD40 ligand (CD40L), CD48 antigen (CD48), SLAM family member 5 (CD84), Complement component C1q receptor (CD93), CD160 antigen (CD160), Scavenger receptor cysteine-rich type 1 protein M130 (CD163), CD166 antigen (CD166), C-type lectin domain family 4 member K (CD207), SLAM family member 7 (CD319), Carcinoembryonic antigen-related cell adhesion molecule 8 (CEACAM8), Chitinase-3-like protein 1 (CHI3L1), Chitotriosidase-1 (CHIT1), C-X-C motif chemokine 1 (CXCL1), C-X-C motif chemokine 13 (CXCL13), C-X-C motif chemokine 16 (CXCL16), C-X-C motif chemokine 17 (CXL17), *E*-selectin, Fc receptor-like B (FcRLB), Growth/differentiation factor 15 (GDF-15), Intercellular adhesion molecule 2 (ICAM-2), ICOS ligand (ICOSLG), Interferon gamma receptor 1 (IFN-γ-R1), Low affinity immunoglobulin gamma Fc region receptor II-b (IgG-FcR-IIb), Interleukin-1 receptor antagonist protein (IL-1ra), Interleukin-1 receptor-like 2 (IL-1RL2), Interleukin-1 receptor type 1 (IL-1RT1), Interleukin-1 receptor type 2 (IL-1RT2), Interleukin-2 receptor subunit alpha (IL-2RA), Interleukin-4 receptor subunit alpha (IL-4RA), Interleukin-6 (IL-6), Interleukin-6 receptor subunit alpha (IL-6RA), Interleukin-17D (IL-17D), Interleukin-17 receptor A (IL-17RA), Interleukin-18 (IL-18), Interleukin-18-binding protein (IL-18BP), Interleukin-27 subunit alpha and beta (IL-27AB), Junctional adhesion molecule A (JAM-A), T-lymphocyte surface antigen Ly-9 (Ly-9), Macrophage receptor MARCO (MARCO), MHC class I polypeptide-related sequence A and B (MIC-A/B), Myeloperoxidase (MPO), P-selectin, Platelet endothelial cell adhesion molecule (PECAM-1), Peptidoglycan recognition protein 1 (PGLYRP1), Polymeric immunoglobulin receptor (PIgR), Pro-interleukin-16 (Pro-IL16), Progranulin, P-selectin glycoprotein ligand 1 (PSGL-1), Pulmonary surfactant-associated protein D (PSP-D), Pentraxin-related protein PTX3 (PTX3), Metalloproteinase inhibitor 4 (TIMP-4), Toll-like receptor 3 (TLR3), Trem-like transcript 2 protein (TLT-2), Alpha-taxilin (TXLNA) and Lymphotactin (XCL1).

## 3 Results

### 3.1 Study population

Characteristics of study population, including sex, age, comorbidities, and medications, are presented in Table 1, as previously described [13]. Patients were followed up for median 5.7 years (IQR 4.6–7.2). Twenty-five patients died during follow-up time and 36 received HT. Atrial fibrillation was less common in the heart transplanted subpopulation (*n* = 7; 37 %) vs patients with LHF-PH (*n* = 37; 55 %). More patients with LHF-PH had diabetes mellitus (*n* = 14; 21 %) and systemic hypertension (*n* = 27; 73 %) vs PreHT (n = 1; 5 %) and (n = 2; 11 %) respectively.

**Table 1:** Demographics of the study population.

Variable	Controls (*n* = 20)		LHF-PH (*n* = 67)		Pre-HT (*n* = 19)		Post-HT (n = 19)	
	n (%)	Median (IQR)	n (%)	Median (IQR)	n (%)	Median (IQR)	n (%)	Median (IQR)
Female	10 (50)		27 (40)		4 (21)		4 (21)	
Age	20 (100)	41 (26.8–50.5)	67 (100)	63 (51–75)	19 (100)	51 (47–62)	19 (100)	52 (49–64)
BSA	19 (95)	1.9 (1.8–2)	67 (100)	1.9 (1.8–2.1)	19 (100)	2 (1.8–2.1)	19 (100)	2 (1.8–2)
BTmean	20 (100)	95 (88.7–99.8)	67 (100)	89.3 (78.7–99.3)	19 (100)	80.7 (77.7–89.7)	19 (100)	100.7 (90.3–106.3)
Creatinine			63 (94)	108 (86–136)	18 (95)	110 (89.5–124.5)	19 (100)	104 (97–121)
eGFR			63 (94)	53.9 (39.6–66.2)	18 (95)	62.8 (54.1–70.5)	19 (100)	61 (46.2–72.7)
HFrEF			36 (54)		18 (95)		–	
HFpEF			31 (46)		1 (5.3)		–	
PH-LHD			67 (100)		19 (100)		–	
Ipc-PH			30 (45)		10 (53)		–	
Cpc-PH			37 (55)		9 (47)		–	
Atrial fibrillation			37 (55)		7 (37)		–	
Diabetes Mellitus			14 (21)		1 (5.3)		6 (32)	
Hypertension			27 (73)		2 (11)		–	

Medication								
Beta-blockers			59 (88)		18 (95)		8 (42)	
Angiotensin converting enzyme inhibitor			30 (45)		9 (47)		19 (100)	
Angiotensin II receptor blocker			–		–		5 (26)	
Mineralcorticoid receptor antagonist			28 (42)		8 (42)		2 (11)	
Furosemide			40 (60)		18 (95)		7 (37)	
Prednisolone			–		–		18 (95)	
Cyclosporine			1 (1.5)		–		2 (11)	
Tacrolimus			–		–		17 (90)	
Mycophenolate myofetil			–		–		15 (79)	
Azathioprine			1 (1.5)		–		3 (16)	

Patient characteristics have previously been described [13]. Patients were excluded before analysis when no heart disease (n = 3) or PH diagnosis (n = 2) were present. PH after HT (n = 1) were excluded from pre- and postHT groups to only be included in LHF-PH. Heart failure with reduced ejection fraction (HFrEF) is defined as an ejection fraction <50 %. Heart failure with preserved ejection fraction (HFpEF) was defined by an ejection fraction ≥50 %.

Abbreviations:

Left sided heart failure with pulmonary hypertension (LHF-PH), before heart transplant (pre-HT), after heart transplant (post-HT), body surface area (BSA), mean blood pressure (BTmean), estimated glomerular filtration rate (eGFR), isolated postcapillary pulmonary hypertension (Ipc-PH), combined postcapillary and precapillary pulmonary hypertension (Cpc-PH), mean arterial pressure (MAP), mean pulmonary arterial pressure (mPAP), mean right arterial pressure (MRAP).

### 3.2 Protein levels of IL-6 and PTX3

Protein levels of all 65 inflammatory proteins in controls, patients with LHF-PH as well as before and after HT are shown in Table 2. Elevated levels of IL-6 and PTX3 were present in LHF-PH compared to controls (*p* < 0.001).

**Table 2:** Protein levels of all 65 inflammatory protein and NT-proBNP.

Biomarker (AU)	Control (*n* = 20)	LHF-PH (n = 67)	PreHT (*n* = 19)	PostHT (n = 19)	*p*-value		
	Median (IQR)	Median (IQR)	Median (IQR)	Median (IQR)	Control vs LHF-PH	Pre-HT vs Post-HT	Control vs post-HT
Annexin A1	2.6 (2.3–3.5)^a^	3.2 (2.7–4)^a^	3.2 (2.8–4.1)^a^	3 (2.7–3.7)	0.0058*	0.7743	0.1041
Azurocidin	2.9 (2.6–3.6)	3.5 (2.9–4.6)	4.8 (3.2–6.3)	3.9 (3.2–5.7)	0.0143	0.5217	0.0084
CCL2	4.6 (3.9–5.3)	6.6 (5.5–7.3)	6.1 (5.3–7.9)	6.9 (5.1–8.6)	<0.0001*	0.4715	0.0002*
CCL3	2.6 (2.2–3.3)	6.2 (5.1–9.8)	5.3 (4.8–10)	4.9 (4.1–6.8)	<0.0001*	0.7381	<0.0001*
CCL15	68 (58–98)	133 (104–204)	124 (104–164)	126 (96–162)	<0.0001*	0.6507	<0.0001*
CCL16	39 (31–48)	61 (44–84)	67 (47–80)	52 (40–67)	0.0001*	0.001*	0.0128
CCL17	135 (70–180)	142 (78–299)	98 (58–217)	76 (50–137)	0.1636	0.4653	0.2244
CCL22	3.7 (3.2–5.6)	3.5 (2.7–5.2)	3.7 (2.3–5.6)	2.6 (1.4–5.1)	0.4846	0.1956	0.0436
CCL24	30 (22–43)	36 (24–56)	25 (20–57)	24 (15–49)	0.2453	0.0663	0.5133
CD27L	146 (132–168)^a^	212 (144–280)^a^	174 (139–232)^a^	172 (125–215)	0.0007*	0.7019	0.4927
CD40-L	86 (56–230)	70 (30–135)	30 (17–87)	36 (19–109)	0.0609	0.1819	0.0043*
CD48	67 (61–78)^a^	79 (63–97)^a^	73 (63–97)^a^	66 (57–86)	0.0302	0.1674	0.977
CD84	40 (34–51)	42 (32–51)	37 (29–43)	37 (32–49)	0.98	0.3124	0.7974
CD93	503 (473–564)	681 (551–864)	681 (601–929)	506 (351–648)	<0.0001*	<0.0001*	0.7705
CD160	19 (17–24)^a^	28 (22–39)^a^	25 (20–35)^a^	28 (20–41)	0.0001*	0.0737	0.0068
CD163	108 (88–135)	132 (110–166)	116 (99–209)	110 (93–143)	0.0079	0.0401	0.6071
CD166	19 (17–22)	22 (18–27)	22 (17–28)	16 (15–19)	0.0047*	<0.0001*	0.0283
CD207	4.4 (3.9–5.3)^a^	5 (4.2–6.1)^a^	5.3 (4.3–6.8)^a^	4.5 (3.4–5.2)	0.0794	0.0665	0.9141
CD319	2.5 (2.2–3.9)	4.3 (3.8–5.6)	4.9 (3.7–5.6)	2.2 (2.2–3.7)	0.0003*	0.001*	0.685
CEACAM8	9.4 (8.2–13)	18 (13–26)	21 (13−30)	18 (14–24)	<0.0001*	0.2753	<0.0001*
CHI3L1	19 (16–24)	54 (31–92)	40 (25–80)	34 (23–46)	<0.0001*	0.0071	0.0001*
CHIT1	2.2 (1.6–3.9)	5.3 (3.1–11)	3.6 (2.7–6.6)	5.6 (2.1–7.3)	<0.0001*	0.0237	0.0074
CXCL1	315 (168–621)	329 (216–491)	228 (132–364)	237 (146–491)	0.8453	0.4653	0.4276
CXCL13	240 (188–325)^a^	412 (343–597)^a^	355 (319–447)^a^	1170 (405–1595)	<0.0001*	0.0013*	<0.0001*
CXCL16	50 (44–55)	59 (52–71)	59 (54–82)	53 (43–60)	0.0003*	0.0001*	0.5315
CXCL17	13 (9.2–23)^a^	43 (33–56)^a^	40 (33–58)^a^	19 (14–37)	<0.0001*	<0.0001*	0.04
E-selectin	3.2 (2.5–3.5)	3.2 (2.5–4.5)	3.8 (2.9–4.5)	2.8 (2.1–3.2)	0.5037	<0.0001*	0.2496
FCRLB	2.2 (1.8–2.7)^a^	3.8 (2.9–5)^a^	3.9 (3–5.4)^a^	2.5 (2.3–2.9)	<0.0001*	0.0001*	0.2146
GDF-15	9.1 (7.2–11)	38 (24–61)	39 (20–73)	28 (13–44)	<0.0001*	0.0108	<0.0001*
ICAM-2	17 (16–23)	23 (17–28)	26 (17–33)	18 (15–22)	0.0141	<0.0001*	0.8786
ICOSLG	11 (9.4–12)^a^	10 (9.3–11)^a^	9.5 (9–11)^a^	10 (9.3–11)	0.3178	0.0814	0.549
IFN-γ-R1	10 (9.8–11)^a^	15 (11–17)^a^	15 (11–17)^a^	12 (11–15)	<0.0001*	0.0101	0.0077
IgG-FcR-IIb	3.3 (2.7–4)	3.1 (2.1–4.4)	2.9 (1.5–4.6)	2.4 (1.8–4.1)	0.7461	0.0817	0.4154
IL-1ra	9.7 (7.8–15)	19 (15–25)	17 (13–26)	17 (14–23)	<0.0001*	0.3525	0.0044*
IL-1RL2	18 (14–24)	23 (18–26)	23 (21–29)	23 (20–25)	0.0197	0.6226	0.0407
IL-1RT1	64 (60–69)	79 (65–104)	87 (67–119)	70 (61–85)	0.0007*	0.0006*	0.1661
IL-1RT2	21 (18–25)	24 (20–28)	25 (22−30)	19 (18–22)	0.124	<0.0001*	0.2217
IL-2RA	9.7 (9.4–11)	13 (10−21)	11 (6.6–22)	8.9 (7.3–12)	0.0004*	0.0003*	0.3469
IL-4RA	4.7 (4.1–5.8)	8.6 (7.4–11)	9.4 (7.8–11)	7.7 (6.2–8.2)	<0.0001*	0.0001*	<0.0001*
IL-6	3.4 (2.3–4.6)^a^	13 (7–19)^a^	13 (8.9–17)^a^	5.6 (3.5–7.6)	<0.0001*	0.0003*	0.0018*
IL-6RA	1438 (1109–1822)	1249 (997–1553)	1107 (890–1726)	1084 (936–1611)	0.0932	0.1042	0.0305
IL-17D	4.6 (4.2–5.3)	7.1 (6.2–8.2)	7 (5.9–8.6)	4.8 (4.3–6.5)	<0.0001*	<0.0001*	0.1596
IL-17RA	12 (8.9–14)	13 (9.9–16)	13 (10–17)	11 (8.6–13)	0.2886	0.0001*	0.5315
IL-18	289 (213–342)	357 (269–434)	347 (237–454)	482 (358–617)	0.0305	0.0082	0.0002*
IL-18BP	44 (42–51)	62 (45–84)	52 (37–70)	51 (40–89)	0.0013*	0.5153	0.2382
IL-27AB	13 (12–15)	23 (20−30)	22 (20−33)	21 (18–26)	<0.0001*	0.0258	<0.0001*
JAM-A	70 (38–90)	62 (50–97)	57 (39–75)	55 (44–72)	0.82	0.6507	0.444
LY9	27 (24–30)^a^	30 (26–35)^a^	29 (25–34)^a^	22 (19–32)	0.0626	0.0898	0.3657
MARCO	60 (56–65)	72 (62–82)	67 (59–77)	73 (62–83)	0.0002*	0.0401	0.001*
MIC-AB	16 (11–18)^a^	19 (15–26)^a^	18 (15–27)^a^	14 (11−20)	0.0079	0.0003*	0.4879
MPO	11 (10−13)	12 (11–15)	13 (11–15)	14 (10–18)	0.1803	0.2413	0.2036
NT-proBNP	1.1 (1.1–1.2)	13 (7.6–32)	28 (17–45)	2 (1.5–6.5)	<0.0001*	<0.0001*	0.0003*
P-selectin	639 (520–1075)	610 (443–849)	491 (403–773)	512 (392–723)	0.333	0.6507	0.0262
PECAM-1	42 (30–51)	40 (30–53)	38 (30–45)	31 (27–37)	0.7436	0.1134	0.0353
PGLYRP1	90 (72–100)	119 (101–170)	114 (83–196)	86 (74–127)	<0.0001*	<0.0001*	0.444
PIgR	48 (45–50)	57 (54–60)	59 (56–63)	54 (51–58)	<0.0001*	0.0124	<0.0001*
Pro-IL16	33 (21–36)	45 (36–57)	46 (33–57)	74 (41–127)	<0.0001*	0.0401	<0.0001*
Progranulin	9.3 (8–9.8)	11 (9.1–13)	11 (8.9–15)	7.5 (6–8.9)	0.0003*	<0.0001*	0.0063
PSGL-1	20 (18–22)	20 (18–22)	21 (17–22)	20 (18–27)	0.5567	0.2579	0.4439
PSP-D	3 (2.6–5.4)	6.2 (4.4–9.1)	8 (4.8–10)	2.5 (2–3.5)	0.0002*	<0.0001*	0.0414
PTX3	6.3 (4.7–7.2)	8 (6.2–11)	10 (7.6–11)	6.2 (5.5–7.6)	0.0009*	<0.0001*	0.5269
TIMP-4	15 (13–17)	22 (18–33)	19 (16–31)	19 (15–23)	<0.0001*	0.1956	0.0083
TLR3	35 (27–43)^a^	35 (24–46)^a^	44 (32–53)^a^	39 (27–51)	0.9292	0.0277	0.3697
TLT-2	16 (14–20)	15 (12–19)	13 (12–19)	15 (13–17)	0.148	0.8596	0.1865
TXLNA	83 (30–127)^a^	64 (34–111)^a^	43 (22–83)^a^	49 (24–92)	0.9958	0.9323	0.297
XCL1	25 (21–29)	58 (39–78)	52 (38–67)	47 (31–55)	<0.0001*	0.1564	0.0004*

The protein levels are expressed in median interquartile range (IQR) in all four groups: control, left heart failure with pulmonary hypertension (LHF-PH), before heart transplant (pre-HT) and after heart transplant (postHT). Plasma samples were analysed using proximity extension assay (PEA). Significant results had *P*-values under 0.006. Protein levels marked with ^a^, had n-1.

Abbreviations: Annexin A1, Azurocidin, C—C motif chemokine 2 (CCL2), C—C motif chemokine 3 (CCL3), C—C motif chemokine 15 (CCL15), C—C motif chemokine 16 (CCL16), C—C motif chemokine 17 (CCL17), C—C motif chemokine 22 (CCL22), C—C motif chemokine 24 (CCL24), CD27 antigen (CD27L), CD40 ligand (CD40L), CD48 antigen (CD48), SLAM family member 5 (CD84), Complement component C1q receptor (CD93), CD160 antigen (CD160), Scavenger receptor cysteine-rich type 1 protein M130 (CD163), CD166 antigen (CD166), C-type lectin domain family 4 member K (CD207), SLAM family member 7 (CD319), Carcinoembryonic antigen-related cell adhesion molecule 8 (CEACAM8), Chitinase-3-like protein 1 (CHI3L1), Chitotriosidase-1 (CHIT1), C-X-C motif chemokine 1 (CXCL1), C-X-C motif chemokine 13 (CXCL13), C-X-C motif chemokine 16 (CXCL16),C-X-C motif chemokine 17 (CXCL17), *E*-selectin, Fc receptor-like B (FcRLB), Growth/differentiation factor 15 (GDF-15), Intercellular adhesion molecule 2 (ICAM-2), ICOS ligand (ICOSLG), Interferon gamma receptor 1 (IFN-γ-R1), Low affinity immunoglobulin gamma Fc region receptor II-b (IgG-FcR-IIb), Interleukin-1 receptor antagonist protein (IL-1ra), Interleukin-1 receptor-like 2 (IL-1RL2), Interleukin-1 receptor type 1 (IL-1RT1), Interleukin-1 receptor type 2 (IL-1RT2), Interleukin-2 receptor subunit alpha (IL-2RA), Interleukin-4 receptor subunit alpha (IL-4RA), Interleukin-6 (IL-6), Interleukin-6 receptor subunit alpha (IL-6RA), Interleukin-17D (IL-17D), Interleukin-17 receptor A (IL-17RA), Interleukin-18 (IL-18), Interleukin-18-binding protein (IL-18BP), Interleukin-27 subunit alpha and beta (IL-27AB), Junctional adhesion molecule A (JAM-A), T-lymphocyte surface antigen Ly-9 (Ly-9), Macrophage receptor MARCO (MARCO), MHC class I polypeptide-related sequence A and B (MIC-A/B), Myeloperoxidase (MPO), P-selectin, Platelet endothelial cell adhesion molecule (PECAM-1), Peptidoglycan recognition protein 1 (PGLYRP1), Polymeric immunoglobulin receptor (PIgR), Pro-interleukin-16 (Pro-IL16), Progranulin, P-selectin glycoprotein ligand 1 (PSGL-1), Pulmonary surfactant-associated protein D (PSP-D), Pentraxin-related protein PTX3 (PTX3), Metalloproteinase inhibitor 4 (TIMP-4), Toll-like receptor 3 (TLR3), Trem-like transcript 2 protein (TLT-2), Alpha-taxilin (TXLNA) and Lymphotactin (XCL1).

### 3.3 Correlations of IL-6 and PTX3

IL-6 correlated to MRAP (*r* = 0.5, *p* < 0.0001), mPAP (*r* = 0.4, *p* = 0.0009) and NT-proBNP (*r* = 0.44, *p* = 0.0002) whereas PTX3 correlated only to NT-proBNP (r = 0.4, *p* < 0.0009). Optimal cut-offs for IL-6 and PTX3 for survival analysis were determined using ROC-curves (survival vs event). IL-6 had an AUC of 0.68 (*p* = 0.038) whereas PTX3 had an AUC of 0.679 (*p* = 0.04).

### 3.4 Prognostic value of IL-6 and PTX3

**Fig. 1:**
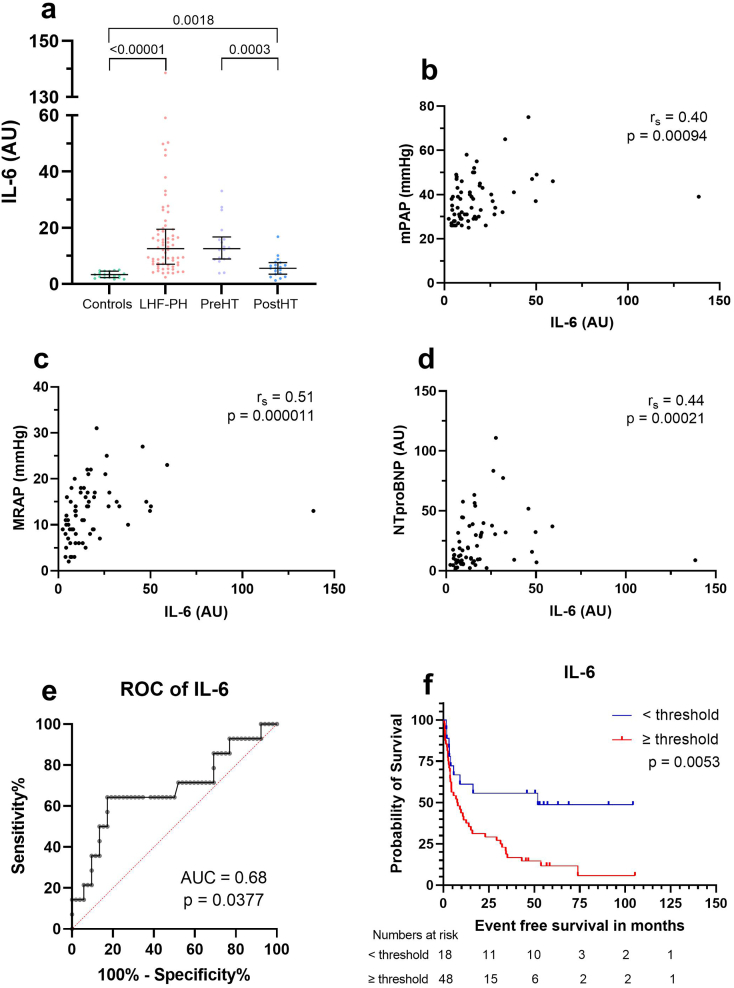
Protein levels of Interleukin 6 (a), Spearman's correlation analyses with haemodynamic parameters and NT-proBNP (b-d), ROC-curve (e) and Kaplan Meier curve (f). (a) Protein levels of interleukin-6 (IL-6) in controls, patients with left heart failure and pulmonary hypertension (LHF-PH), before heart transplantation (PreHT) and after heart transplant (PostHT). (b-d) Correlation analysis between IL-6 and (b) mean pulmonary arterial pressure (mPAP), (c) mean right atrial pressure (MRAP), and (d) N-terminal pro brain natriuretic peptide (NT-proBNP). (e) ROC-curve of IL-6 to determine threshold for further analysis. (f) Kaplan Meier analysis of patients with LHF-PH (*n* = 67) dichotomized accordance with attained IL-6 threshold from receiver operating characteristics analysis. During the follow-up period of 48 patients with LHF-PH with higher IL-6 levels exhibited worse survival.

**Fig. 2:**
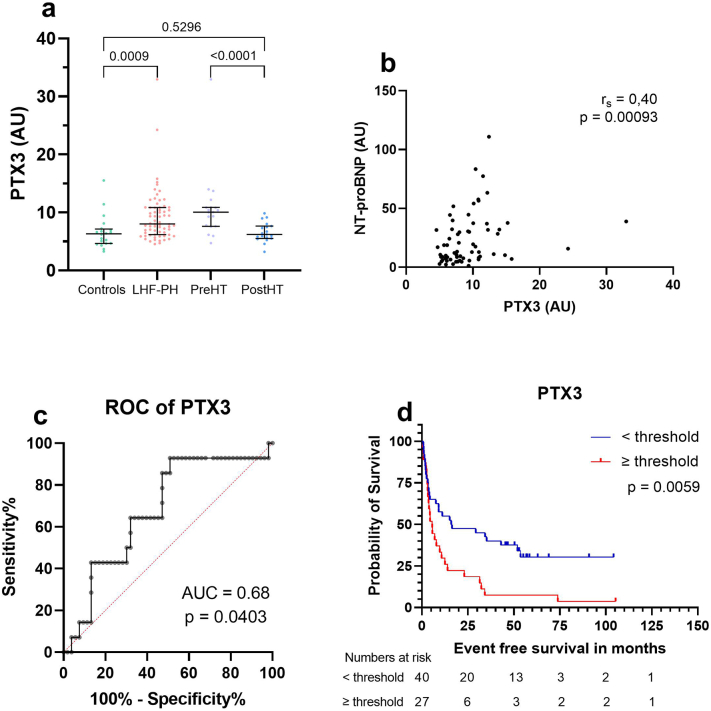
Protein levels of PTX3 (a), Spearman's correlation analyses with NT-proBNP (b), ROC-curve (c) and Kaplan Meier (d). (a) Protein levels of Pentraxin related protein PTX3 (PTX3) in controls, patients with left heart failure and pulmonary hypertension (LHF-PH), before heart transplantation (PreHT) and after heart transplant (PostHT). (b) Correlation analysis between PTX3 and N-terminal pro brain natriuretic peptide (NT-proBNP). (c) ROC-curve of PTX3 to determine threshold for further analysis. (d) Kaplan Meier analysis of patients with LHF-PH (n = 67) dichotomized accordance with attained PTX3 threshold from receiver operating characteristics analysis. During the follow-up period of 27 patients with LHF-PH with higher PTX3 levels exhibited worse survival.

In LHF-PH, survival analysis with Kaplan Meier showed that higher plasma levels of IL-6 and PTX3 were associated with worse survival (*p* < 0.01) (Fig. 1, Fig. 2). No other protein had any significant results in survival analysis, and therefore this study focuses on IL-6 and PTX3 as the two proteins shows the best prognostic value.

**Fig. 3:**
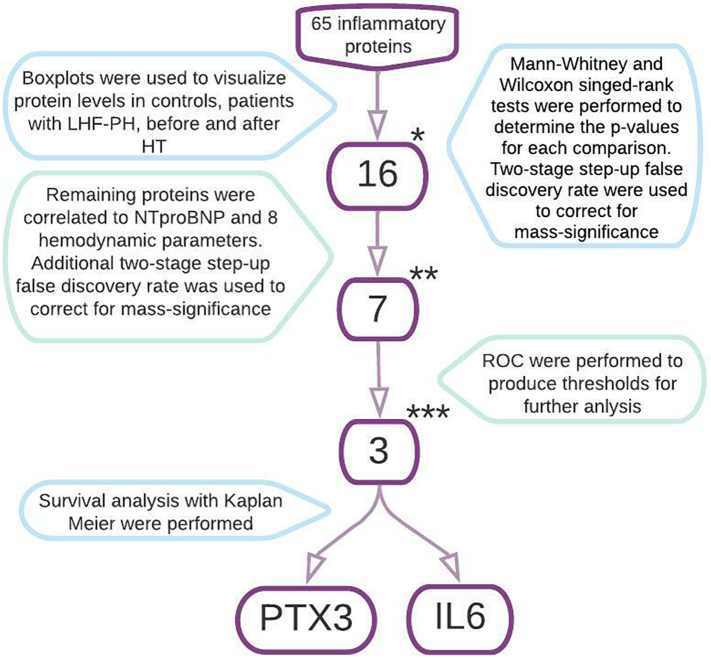
Study design explained. Statistical significance for boxplots were *p* < 0.006 and false discovery rate was performed with Q = 0.01 * Proteins included in correlation analysis were IL-6, CXL17, FCRLB, IL2-RA, ALCAM, GRN, CXCL16, PSP-D, IL-1RT1, PGLYRP1, CD93, CCL16, SLAMF7, IL-4RA, IL-17D, PTX3. ** Proteins included in analysis with ROC-curves were IL-6, CXL17, GRN, IL-1RT1, CD93, IL4RA, PTX3. *** Proteins included in Kaplan Meier analysis were IL-6, IL-4RA, PTX3. For a full list of protein abbreviations, please see Section 2.5 “Proteins” in Methods. Abbreviations: Left heart failure with pulmonary hypertension (LHF-PH), heart transplantation (HT), N-terminal pro brain natriuretic peptide (NT-proBNP), Interleukin 6 (IL-6), pentraxin related protein PTX3 (PTX3).

### 3.5 Correlations between other proteins and haemodynamic parameters or NT-proBNP

IL-6, PTX3, CXCL17, progranulins, IL-1RT1, CD93 and IL-4RA showed at least one significant correlation to haemodynamic parameters or NT-proBNP (supplementary Table 1). The strongest correlation was between IL-1RT1 and NT-proBNP (*r* = 0.61, *p* < 0.0001).

### 3.6 Subgroup analysis

In the subgroup analysis, in LHF-PH, higher levels of PTX3 were found in those taking ACEi vs those who did not (*p* = 0.03). Higher levels of PTX3 were also found in patients Post-HT with than without DM(*p* = 0.007).

## 4 Discussion

Better risk stratification and mortality predictions may be made when combining different modalities, including haemodynamic- and clinical parameters with biomarkers [9,14,15]. The present study found that the inflammatory mediators IL-6 and PTX3 were elevated in plasma in patients with LHF-PH and decreased after HT. We also demonstrated that higher levels of IL-6 and PTX3 were associated with worse survival in patients with LHF-PH. Moreover, IL-6 correlated with multiple haemodynamic parameters including NT-proBNP, whereas PTX3 only with NT-proBNP.

IL-6 is an inflammatory cytokine that is released from immune cells and stromal cells [15,16] in response to antigen stimulation of pattern recognition receptors [16]. IL-6 is involved in leukocyte switch, release of acute phase reactants from the liver [16] and induces fibroblast proliferation and synthesis of extracellular matrix [17] which are key processes in HF progression [6]. IL-6 has previously been reported elevated in PH without LHF, however, with no correlation to survival [14]. In HF, elevation of IL-6 has been found to predict survival [18]. Further, in a murine model of PH-LHD, it has been suggested that IL-6 signaling pathway is activated [19] and elevated IL-6 levels have also been shown to correlate with lower quality of life, worse right ventricular function and stroke volume in patients with pulmonary arterial hypertension (group 1 PH) [20]. In line with above mentioned studies, we found that IL-6 were elevated in patients with LHF-PH compared to controls, with these levels decreasing one-year after HT and associated to worse survival in patients with LHF-PH. This is further supported by the positive correlations to NT-proBNP which is elevated in HF, mPAP which is used to diagnose PH and MRAP which can be elevated in right heart failure or severe PH. Theoretically, given the elevated levels of IL-6 in LHF-PH, and its function in inflammatory activation of fibrosis and HF progression, it may be suggested that IL-6 activates fibrosis and remodeling in LHF-PH as well.

PTX3 is an acute phase reactant that is produced locally by stromal cells and myeloid cells [15,21] and induced by other inflammatory components like IL-1beta, TNF-alpha, TLR, LPS or flagellin [15]. We showed elevated levels of PTX3 in patients with LHF-PH and a decrease in protein levels after HT. Additionally, higher levels of PTX3 were associated with worse prognosis in patients with LHF-PH, which were also supported by the positive correlation of PTX3 and the heart failure biomarker NT-proBNP. This is in line with previous studies where elevated levels of PTX3 have been related to more hospitalization and death in patients with chronic HF (CHF) [22,23], as well as to worse prognosis in HF [15]. Moreover, PTX3 has also been identified as an independent risk factor and predictor of cardiac events and death in patients with CHF [[24], [25], [26]]. Subgroup analysis indicated that out of IL-6 and PTX3 only PTX3 levels in the LHF-PH and PostHT group were independent of comorbidities. All groups could, however, not be included in the analyses due to low number or no participant taking ACEi or having DM or HTN.

Current limitations of this study include the relatively small patient population as well as a control group that is younger than patients with LHF-PH. This could potentially interfere with our results as both IL-6 and PTX3 have been previously proven elevated in older people and PTX3 also higher in women than in men [18,23]. Further, elevated PTX3 have also been reported in people with ventricular dysfunction, atrial fibrillation, and diabetes mellitus [15] which are comorbidities present in our study population. In addition, the impact of medications, including immunosuppressive therapy needs to be investigated in future studies. Strengths of the present study include the use of invasively measured haemodynamics and the use of PEA, which confers high specificity and sensitivity in protein measurement. FDR was also used to correct for mass-significance given the large number of statistical tests used.

In conclusion, we found elevated plasma levels of IL-6 and PTX3, each of which were associated with worse survival in patients with LHF-PH. The results are in line with previous research and present an interesting approach for new prognostic biomarkers to be used clinically in patients with LHF-PH. Future studies are needed to validate our results in larger cohorts as well as investigate the impact of age, demographics, and comorbidities on the proteins' levels.

## CRediT authorship contribution statement

Sara Helleberg: conceptualization, methodology, software, investigation, writing – original draft, writing – review & editing, formal analysis, visualization.

Adam Engel: software, writing – review & editing, visualization.

Salaheldin Ahmed: conceptualization, methodology, investigation, data collection, writing – review & editing, supervision, project administration.

Abdulla Ahmed: conceptualization, methodology, investigation, data collection, writing – review & editing, supervision and project administration.

Göran Rådegran: conceptualization, methodology, investigation, data collection, resources, writing – review & editing, supervision, project administration, and funding acquisition. All authors are accountable for all aspects of the work and approve of the final version to be published.

## Declaration of competing interest

The authors declare the following financial interests/personal relationships which may be considered as potential competing interests: Göran Rådegran reports financial support was provided by Janssen Cilag AB. Göran Rådegran reports financial support was provided by Avtal om läkarutbildningen och forskning, ALF. Salaheldin Ahmed reports a relationship with Janssen Cilag AB that includes: speaking and lecture fees. Abdulla Ahmed reports a relationship with Janssen Cilag AB that includes: speaking and lecture fees. Göran Rådegran reports a relationship with Janssen Cilag AB that includes: consulting or advisory, funding grants, and speaking and lecture fees. Göran Rådegran reports a relationship with Actelion Pharmaceuticals Sverige AB that includes: speaking and lecture fees. Göran Rådegran reports a relationship with Bayer health care that includes: speaking and lecture fees. Göran Rådegran reports a relationship with GlaxoSmithKline that includes: speaking and lecture fees. Göran Rådegran reports a relationship with Nordic infucare that includes: speaking and lecture fees. Dr. Rådegran is, and has been primary-, or co-, investigator in; clinical PAH trials for Acceleron, Actelion Pharmaceuticals Sweden AB, Bayer, GlaxoSmithKline, Janssen, Pfizer, and United Therapeutics, and in clinical heart transplantation immunosuppression trials for Novartis. The companies had no role in the data collection, analysis and interpretation and had no right in disapproving of the manuscript.

## Figures and Tables

**Supplementary Fig. 1:**
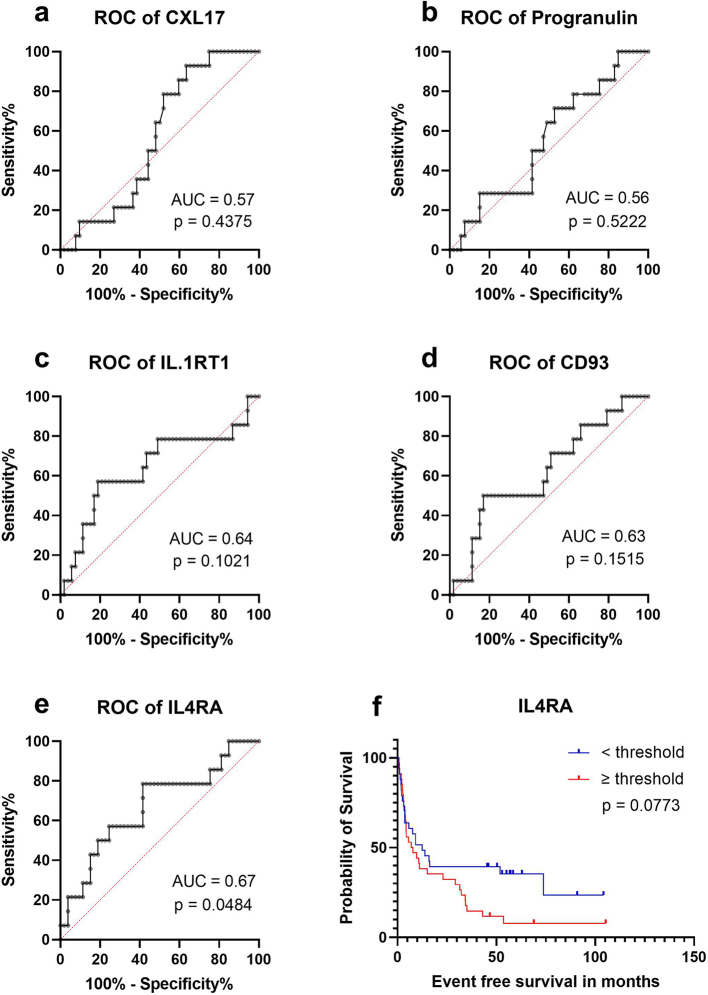
ROC curves (a-e) and Kaplan Meier curves (f) for proteins included in the analysis other than Interleukin 6 and Pentraxin related protein 3. For ROC-and Kaplan Meier curves for IL-6 and PTX3 please see Fig. 1, Fig. 2 respectively.

## Appendix A Supplementary data

Supplemantary Tables and Figure Legends. Image 1
