# Supplementary material for: Higher plasma IL-6 and PTX3 are associated with worse survival in left heart failure with pulmonary hypertension
Source: Am Heart J Plus. 2022 Aug 10;20:100190. doi: 10.1016/j.ahjo.2022.100190 (PMC10978361; doi:10.1016/j.ahjo.2022.100190)
Supplement: Supplementary file 1 — Supplemantary Tables and Figure Legends. [file mmc2.docx]

| **Supplementary Table 1** **Spearmans´Correlation Analysis of 16 Inflammatory Proteins with 8 Hemodynamic Parameters and NT-proBNP** | | | | | | | | | | |  |
| --- | --- | --- | --- | --- | --- | --- | --- | --- | --- | --- | --- |
| Variables | mPAP | PAWP^a^ | MRAP | SVI | CI | PVR^a^ | LVSWI^a^ | PAC | NT-proBNP | | |
| CCL16 | 0.05 (0.7029) | -0.06 (0.6115) | 0.23 (0.0664) | -0.07 (0.5942) | 0.01 (0.9234) | -0.01 (0.9059) | -0.08 (0.519) | 0.23 (0.065) | 0.24 (0.0538) | | |
| CD93 | 0.25 (0.0421) | 0.17 (0.1808) | 0.28 (0.0242) | -0.12 (0.3459) | -0.02 (0.8835) | 0.22 (0.0732) | -0.18 (0.1408) | -0.13 (0.2843) | 0.46 (0.0001*) | | |
| CD166 | 0.38 (0.0016) | 0.09 (0.455) | 0.37 (0.0018) | -0.08 (0.5062) | 0.06 (0.6439) | 0.35 (0.004) | -0.02 (0.862) | -0.22 (0.0776) | 0.27 (0.0274) | | |
| CD319 | 0.22 (0.0758) | 0.13 (0.31) | 0.4 (0.0029) | 0.06 (0.6029) | 0.03 (0.8116) | 0.07 (0.5666) | -0.02 (0.8712) | 0.14 (0.2712) | 0.4 (0.0023) | | |
| CXCL16 | 0.3 (0.0153) | 0.16 (0.1895) | 0.38 (0.0017) | -0.08 (0.5075) | 0.03 (0.82) | 0.20 (0.1009) | -0.04 (0.7715) | -0.2 (0.1065) | 0.38 (0.0017) | | |
| CXCL17^a^ | 0.44(0.0002*) | 0.15 (0.2235) | 0.24 (0.0554) | -0.07 (0.6004) | -0.02 (0.874) | 0.40 (0.0009*) | -0.02 (0.8962) | -0.37 (0,0024) | 0.32 (0.008) | | |
| FCRLBa | 0.14 (0.2613) | -0,003 (0,9801) | 0.24 (0.0498) | -0.02 (0.895) | 0.06 (0.6352) | 0.04 (0.7462) | -0.04 (0.7465) | 0.08 (0.5109) | 0.29 (0.0187) | | |
| IL-1RT1 | 0.31 (0.0103) | 0.2 (0.1119) | 0.55 (<0.0001*) | -0.22 (0.0802) | -0.12 (0.3175) | 0.22 (0.0829) | -0.26 (0.0374) | -0.12 (0.3373) | 0.61 (<0.0001*) | | |
| IL2-RA | 0.33 (0.0064) | -0.09 (0.4795) | 0.21 (0.085) | 0.14 (0.2463) | 0.2 (0.1051) | 0.32 (0.0089) | 0.15 (0.24) | -0.27 (0.029) | 0.14 (0.2678) | | |
| IL-4RA | 0.37 (0.0023) | 0.13 (0.3089) | 0.42 (0.0005*) | -0.11 (0.3926) | 0.03 (0.7808) | 0.32 (0.009) | -0.12 (0.3554) | -0.21 (0.0819) | 0.45 (0.0001*) | | |
| IL-6^a^ | 0.40 (0.0009*) | 0.26 (0.0335) | 0.51 (<0.0001*) | -0.23 (0.0629) | -0.06 (0.6548) | 0.16 (0.2052) | -0.20 (0.1039) | -0.17 (0.1805) | 0.44 (0.0002*) | | |
| IL-17D | 0.25 (0.0412) | -0.07 (0.5944) | 0.08 (0.5106) | 0.16 (0.1876) | 0.18 (0.1482) | 0.34 (0.0047) | 0.19 (0.1198) | -0.22 (0.0738) | 0.13 (0.308) | | |
| PGLYRP1 | 0.17 (0.1659) | -0.09 (0.4795) | 0.19 (0.1318) | -0.04 (0.7441) | 0.12 (0.3164) | 0.24 (0.0539) | -0.02 (0.8858) | -0.01 (0.9242) | 0.24 (0.0504) | | |
| Progranulin | 0.25 (0.0391) | 0.11 (0.4335) | 0.51 (<0.0001*) | -0.18 (0.1345) | -0.17 (0.1693) | 0.25 (0.0475) | -0.22 (0.0738) | -0.02 (0.8494) | 0.47 (<0.0001*) | | |
| PSP.D | 0.11 (0.3943) | 0.0009 (0.9944) | -0.11 (0.3966) | -0.24 (0.0496) | -0.28 (0.0195) | 0.24 (0.056) | -0.21 (0.09) | -0.31 (0.0115) | 0.18 (0.1382) | | |
| PTX3 | 0.19 (0.1242) | 0.13 (0.2884) | 0.21 (0.0842) | -0.14 (0.2609) | -0.09 (0.4487) | -0.01 (0.909) | -0.15 (0.2207) | 0.04 (0.7778) | 0.4 (0.0009*) | | |
|  | | | | | | | | | | | |
|  | | | | | | | | | |  |  |

Each value corresponds to an r(Spearman´s correlation coefficient) (p-value).
^a^ indicates one missing value. * Indicates significant result
Protein levels (and NT-proBNP) are expressed in linear normalized protein expression scale in arbitrary units (AU). Statistical significance after FDR (Q=0.01) were p<0.00096.
mPAP, PAWP, MRAP were measured in mmHg. PVR was measured in wood units.
Abbreviations: C-C motif chemokine 16 (CCL16), Complement component C1q receptor (CD93), CD166 antigen (CD166), SLAM family member 7 (CD319), cardiac index (CI), C-X-C motif chemokine 16 (CXCL16), C-X-C motif chemokine 17 (CXCL17), Fc receptor-like B (FcRLB), Interleukin-1 receptor type 1 (IL-1RT1), Interleukin-2 receptor subunit alpha (IL-2RA), Interleukin-4 receptor subunit alpha (IL-4RA), Interleukin-6 (IL-6), Interleukin-17D (IL-17D), left ventricular stroke work index (LVSWI), mean pulmonary arterial pressure (mPAP), mean right atrial pressure (MRAP), N-terminal pro brain natriuretic peptide (NT-proBNP), pulmonary arterial compliance (PAC), pulmonary arterial wedge pressure (PAWP), Peptidoglycan recognition protein 1 (PGLYRP1), Pulmonary surfactant-associated protein D (PSP-D), Pentraxin-related protein PTX3 (PTX3), pulmonary vascular resistance (PVR), stroke volume index (SVI).

| **Supplementary Table 2 Patient’s Haemodynamic Parameters** | | | |  |
| --- | --- | --- | --- | --- |
| Variable | LHF-PH (n=67) | PreHT (n=19) | PostHT (n=19) | |
|  | Median (IQR) | Median (IQR) | Median (IQR) | |
| MAP | 89 (79 - 99) | 82 (78 - 90) | 101 (90 - 106) | |
| sPAP | 49 (43 - 65) | 47 (40 - 57) | 23 (18 - 27) | |
| dPAP | 24 (20 - 29) | 24 (23 - 29) | 8 (6 - 11) | |
| mPAP | 34 (29 - 43) | 31 (29 - 39) | 13 (12 - 17) | |
| PAWP | 22 (18 - 26)^a^ | 23 (19 - 27)^a^ | 6 (4 - 8) | |
| MRAP | 13 (8 - 16) | 14 (9 - 17) | 2.5 (0 - 4)^a^ | |
| HR | 70 (65 - 84) | 73 (69 - 78) | 81 (73 - 87) | |
| CO | 3.7 (3 – 4.6) | 3.2 (2.6 - 4) | 5.4 (4.9 – 6.5) | |
| AV.O2diff | 61 (51 - 79) | 74 (69 - 82) | 42 (40 - 51)^b^ | |
| SaO2 | 95 (92 - 96) | 96 (93 - 96) | 97 (96 - 98)^b^ | |
| SvO2 | 56 (48 - 63) | 49 (46 - 57) | 70 (67 - 72) | |
| SV | 50 (42 - 63) | 45 (34 - 59) | 72 (66 - 78) | |
| SVI | 27 (22 - 34) | 23 (18 - 29) | 36 (34 - 39) | |
| CI | 1.9 (1.5 – 2.4) | 1.6 (1.4 – 2.1) | 2.9 (2.6 – 3.2) | |
| PVR | 3.4 (2.4 – 4.1)^a^ | 3.2 (2.3 – 3.6)^a^ | 1.4 (0.9 – 1.9) | |
| PVRI | 1.7 (1.2 – 2.3)^a^ | 1.7 (1.2 – 1.8)^a^ | 0.69 (0.51 - 1) | |
| RVSWI | 643 (385 - 888) | 389 (303 - 725) | 432 (320 - 524)^a^ | |
| LVSWI | 1850 (1133 - 2510)^a^ | 1461 (1028 - 1805)^a^ | 3278 (3186 - 3876) | |
| PAC | 1.8 (1.4 – 2.8) | 1.8 (1.7 – 3.1) | 5.1 (4 – 6.3) | |

**Levels of haemodynamic parameters** expressed in median(interquartile range). Cells marked with ^a^ were missing one value which equals n-1 and cells marked with ^b^ were missing two values, n-2

Abbreviations: arterial venous oxygen saturation difference (AV O2diff), cardiac index (CI), cardiac output (CO), diastolic pulmonary arterial pressure (dPAP), heart rate (HR), left sided heart failure with pulmonary hypertension (LHF-PH), left ventricular stroke work index (LVSWI), mean arterial pressure (MAP), mean pulmonary arterial pressure (mPAP), mean right atrial pressure (MRAP), pulmonary arterial compliance (PAC), pulmonary arterial wedge pressure (PAWP), after heart transplant (post-HT), before heart transplant (pre-HT), pulmonary vascular resistance (PVR), pulmonary vascular resistance index (PVRI), right ventricular stroke work index (RVSWI), arterial oxygen saturation (SaO_2_), systolic pulmonary arterial pressure (sPAP), stroke volume (SV), stroke volume index (SVI), mixed venous oxygen blood saturation (SvO_2_).

| **Supplementary table 3** | | | | |
| --- | --- | --- | --- | --- |
|  |  | ACEi | DM | HTN |
| IL-6 | LHF-PH | 0,916 | 0,7833 | 0,137 |
|  | PreHT | 0,6665 |  |  |
|  | PostHT |  | 0,6388 |  |
|  | Controls |  |  |  |
| PTX3 | LHF-PH | 0,0343* | 0,272 | 0,7582 |
|  | PreHT | 0,447 |  | 0,2924 |
|  | PostHT |  | 0,0066* |  |
|  | Controls |  |  |  |

**Subgroup analysis with Mann Whitney rank test expressed in p-values for each participant group for the different proteins. Empty cells had few events so analysis could not be performed.
*determines statistical significance, p<0,05**

**Abbreviations: Angiotensin converting enzyme inhibitors (ACEi), Diabetes Mellitus (DM), hypertension (HTN), Interleukin 6 (IL-6), Left sided heart failure with pulmonary hypertension (LHF-PH), before heart transplantation (PreHT), after heart transplantation (PostHT), pentraxin related protein 3 (PTX3).**

**Supplementary figure 1. ROC curves (a-e) and Kaplan Meier curves (f) for proteins included in the analysis other than Interleukin 6 and Pentraxin related protein 3.
For ROC-and Kaplan Meier curves for IL-6 and PTX3 please se figures 1 and 2 respectively.**

**Abbreviations: Area under the curve (AUC), Complement component C1q receptor (CD93), C-X-C motif chemokine 17 (CXL17), Interleukin-1 receptor type 1 (IL-1RT1), Interleukin-4 receptor subunit alpha (IL-4RA).**
